# Gambling-related suicides and suicidality: A systematic review of qualitative evidence

**DOI:** 10.3389/fpsyt.2022.980303

**Published:** 2022-10-26

**Authors:** Virve Marionneau, Janne Nikkinen

**Affiliations:** Faculty of Social Sciences, Centre for Research on Addiction, Control, and Governance, University of Helsinki, Helsinki, Finland

**Keywords:** gambling, suicide, qualitative, review, indebtedness, shame

## Abstract

The relationship between gambling and suicides or suicidality has received much research attention in recent years. Review studies have already mapped the quantitative occurrence of suicide attempts, suicides, and self-harm among gamblers, and found a positive association. Related comorbidities and conditions have also been charted in previous reviews. However, there is still a gap in knowledge regarding the actual processes that connect gambling and suicidal behavior. To understand these processes, the current paper conducts a systematic review of qualitative evidence on gambling-related suicides and suicidality. The aim was to identify the role of gambling as well as of confounding factors in suicidality, and what kind of support suicidal individuals have received or would need. We searched for relevant literature in seven scientific databases. We included all studies that presented empirical qualitative evidence on gambling-related suicide, suicidality and/or self-harm (*N* = 20). The results show two main processes that connect gambling and suicidal behavior: indebtedness and shame. At the same time, suicide is a multifactorial phenomenon, and related to other confounding factors. These include psychiatric conditions, personality traits, and life conditions. In many cases, these appear to emerge as a consequence of gambling. Treatment for suicidality has been effective in some cases, but indebtedness and shame may also function as barriers to help-seeking. We conclude that effective prevention is needed by adapting a more comprehensive public health approach and population-level interventions.

## Introduction

Gambling is a serious public health issue that is connected to a multitude of harms to individuals, communities, and societies. Suicide is among the most severe harmful consequences that have been connected to gambling (1–3). The relationship between gambling and suicide has evoked ample research interests recently, with several reviews published on the topic. Two recent scoping reviews (1, 4) as well as earlier reviews (5, 6) have mapped the quantitative occurrence of suicide attempts, suicides, and other self-harm among gamblers. These have found a positive association between gambling and suicidality. Related comorbidities or conditions have also been systematically mapped in young people (7) and gamblers more generally (2, 4). While knowledge on the prevalence of gambling-related suicides has increased, the existing reviews have also identified a gap in understanding the actual process that connects gambling and suicidality (2, 4, 8). To better understand this connection, the current paper conducts a review of qualitative evidence on gambling-related suicides and suicidality.

Quantitative evidence suggests that suicidality is high among those who gamble at harmful levels. In clinical populations and in treatment services for problem gambling, between 22 and 81 percent of individuals have been found to have suicidal ideations, while between 7 and 30 percent of individuals have had suicide attempts (9–16). In other reporting, approximately 40 percent of help-seekers have been identified as presenting a risk of suicide (17). In community samples, between 17 and 39 percent of those who gamble problematically have been reported to have suicide ideation and between 2 and 57 percent have reported suicide attempts (18–22). Suicidality also appears to increase alongside the severity of gambling-related problems (11, 14, 23, 24).

Suicidality amongst those who gamble at problematic levels is notably higher than in the general population. In Italy, the incidence rate ratio for suicide among those diagnosed with gambling disorder was 93.72 compared to the general population (18). In Sweden, individuals with diagnosed gambling disorder have been shown to have a 1.8-fold increase in mortality and a 15-fold increase in suicide mortality compared to the general population (25). Based on another Swedish population-based study, suicidal attempts were twice as common among those who gamble problematically (6.6 percent) compared to controls (3.3 percent). Suicidal ideations were almost twice as common among those who gamble problematically (21.2 percent) compared to controls (11.2 percent) (26). In a UK population study, 19.2 percent of problem gamblers had thought about suicide in the past year, in comparison to 4.1 percent among those with no signs of problem gambling. In the same study, 4.7 percent of problem gamblers had made a suicide attempt in the past year, in comparison to 0.6 percent of those with no problem gambling (24). Some population groups, such as the young, are particularly vulnerable (3, 27). In the UK, the adjusted odds ratio for attempted suicide for young men with problem gambling was 9.0, and 4.9 for young women with problem gambling (27).

The connection between gambling and suicidality has also been studied using coroner’s files as well as comparative statistics between casino and non-casino communities. Research utilizing coroner’s court files in Hong Kong has shown that approximately 20 percent of cases show evidence of gambling prior to death (28). Many of these have been connected to gambling-related indebtedness (28, 29). In comparative statistical work, Phillips et al. (30) has shown that Las Vegas displays the highest levels of suicide in the United States, for both residents and visitors. In Atlantic City, suicide levels also increased after casinos were opened. However, others have argued that gambling is not the most likely reason for the suicide in most cases even in Las Vegas (31) and that statistically suicide rates do not differ between casino and control communities (32). Any increases in suicidality within casino areas have rather been attributed to tourism (33).

The causal link between gambling and suicide has also been challenged by some authors. It has for example been suggested that suicide may not be directly linked to the severity of gambling, but to related depression, indebtedness, or to the stress and anxiety resulting from gambling (34, 35). Blaszczynski and Farrell (36) have also suggested that research on gambling-related suicide is burdened with problems of definition and confusion between suicidality and non-suicidal self-inflicted injuries and passive thoughts of escape.

The close association between suicidal risk and diagnosed pathological or disordered gambling may at least partly be explained by common underlying risk factors or diagnoses. Personality characteristics, such as impulsivity or poor coping skills (17, 37) or life situations such as trauma (17), loss of family relationships or home (38), family conflict (39), or a family history of addiction (40) have each been connected to the co-occurrence of gambling and suicidality. Furthermore, mood disorders, anxiety, and substance use disorders have been associated with suicidal ideation among diagnosed pathological gamblers (10, 17, 25, 38, 41–44).

In Sweden, a register-based study of individuals diagnosed with disordered gambling found that suicidal behavior was significantly associated with mood disorders (OR 2.65), anxiety disorders (OR 1.78), alcohol use disorder (OR 1.95), and drug use disorder (OR 3.6) (42). Co-occurring substance use has also been shown to distinguish between individuals who had suicide attempts instead of ideation only (22) and to increase the odds of suicidality (42, 45). It must be noted, however, that the co-occurrence of other diagnoses does not conclusively negate the role of gambling in suicidal behavior because the temporal sequencing between for example depression and gambling is also not evident (46). It is also possible that gambling is at the root of mood disorders. Other research also indicates that rates of suicidality are higher among problem and pathological gamblers also when levels of depression or alcohol dependence are controlled (3, 35).

Quantitative approaches to study gambling-related suicidality are important from an epidemiological perspective but leave gaps in our knowledge regarding qualitative insight into the processes that connect gambling and suicidality (44, 47). These gaps are not only characteristic of gambling literature, but a more general trend in suicide research. Research on suicide has for long been dominated by quantitative approaches focusing on risk factors and prevalence estimates. Hjelmeland and Knizek (48) have found that between 2005 and 2008, only three percent of studies published in leading international suicide research journals used a qualitative methodology.

In the current study, we focus on determining how gambling contributes to suicides and suicidality based on a systematic review of qualitative evidence on the relationship between gambling and suicide or suicidality. A qualitative systematic review methodology [e.g., Seers (49)] sheds light on what the role of gambling as well as confounding factors are in suicides and what kind of needs suicidal individuals would have for treatment and support.

## Materials and methods

We conducted a systematic review of qualitative studies addressing gambling-related suicidality and suicides. Following the qualitative systematic review methodology as described by Seers (49), the aim was to synthesize qualitative evidence and to understand and interpret the “why” and “how” behind the association between gambling and suicidality or suicide. The research question informing the analysis process was *how gambling contributes to suicides and suicidality and what is the relationship between the two*.

First, we identified relevant studies by conducting a literature search in eight scientific databases: Scopus, PubMed, Ebscohost, ProQuest, Core, OpenAire, Web of Science, and Google Scholar. The search focused on academic, peer-reviewed research articles and books that mainly focused on qualitative research and case study reports on gambling-related suicides and suicidality. This excluded gray literature such as reports as well as anecdotal evidence and quantitative research. The keywords used to conduct the searches were gambling AND suicide AND qualitative OR case study. Articles in any language were included at this stage. We did not limit the search to specific years, but included all references published by May 2022.

**FIGURE 1:**
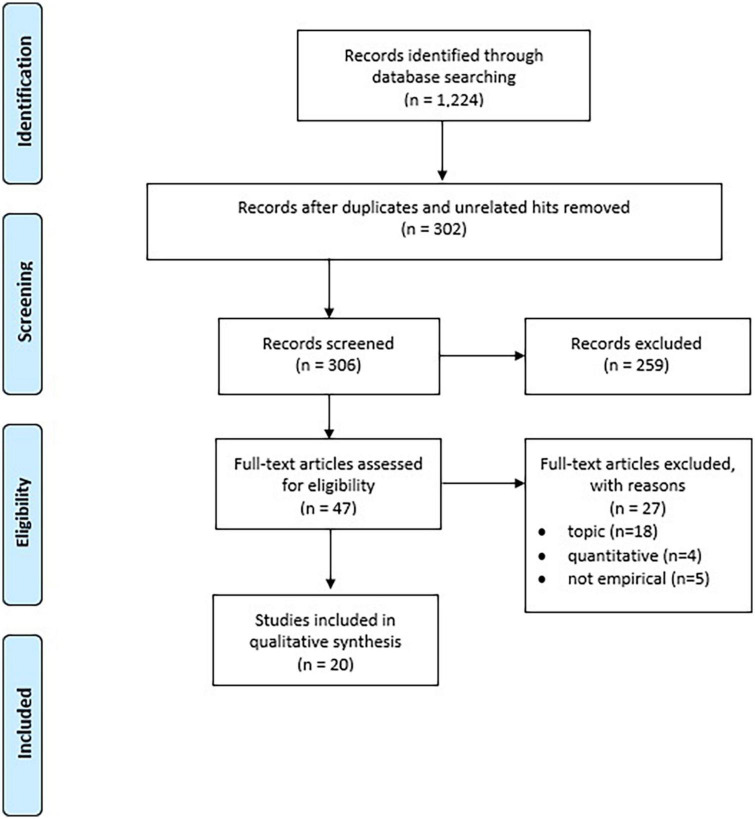
Prisma flow chart.

We then moved on to study selection. Figure 1 is adapted from the PRISMA guidelines of the inclusion process (50, 51). Our search yielded a total of 1,224 records. Based on the titles of references, we first removed the duplicates and unrelated hits (*N* = 918). We then screened the remaining 306 records for their relevance based on their abstracts, resulting in the exclusion of a further 259 papers. The inclusion criteria for this study were that the study would present empirical qualitative evidence on gambling-related suicide, suicidality, or self-harm. Due to the scarcity of qualitative research on the topic, we also included studies that were not mainly focused on gambling-related suicides or suicidality but did include empirical evidence on this topic. These included studies focusing more generally on suicidality or on gambling harms. The corresponding exclusion criteria at this stage were: (1) papers that only included quantitative evidence (*N* = 66); (2) papers that did not include empirical evidence, including reviews and protocols (*N* = 24); (3) papers written in a language we could not understand (not applicable as all records were in English, French, or Spanish); (4) papers with a topic not related to gambling-related suicide, suicidality, and self-harm (*N* = 163); (5) papers focusing on the suicidality of concerned significant others of gamblers instead of gamblers (*N* = 2); and (6) unfound papers (*N* = 4).

The remaining 47 papers were then read through to determine whether they qualified based on our inclusion criteria. A further 27 papers were excluded at this stage either because their focus was not on gambling-related suicidality (*N* = 18), they only included quantitative evidence (*N* = 4), or they did not include empirical evidence (*N* = 5). The final sample consists of 20 empirical research papers. The final sample, including reference, data, and funding source, is described in Table 1.

**TABLE 1:** Studies included in the review.

References	Context	Data	Funding	Causality	Confounding factors	Treatment
Anderson et al. (55)	Estonia	Case study, psychological autopsy of a familicide-suicide	Estonian Ministry of Defense	Perpetrator suffered from pathological gambling. Did not exhibit suicidal behavior before commencing heavy gambling on EGMs.	Impulsive personality trait, HIV diagnosis resulting in marital trouble. Major depression and suicide attempt after gambling commenced.	Medication for depression
Bai et al. (57)	China (rural)	In-depth interviews with CSOs of 242 individuals who died by suicide	American Foundation for Suicide Prevention; Natural Science Foundation of Guanxi Zhuan autonomous region (China)	In one case suicide was attributed to accumulated gambling debts.	n/a	n/a
Balhara et al. (53)	India	Online media reporting on gaming-related suicide (including gambling)	None	94.8 percent of reports directly related suicide to gaming; 83.3 percent of reports linked suicide to financial loss due to gaming (of which 44 percent gambling losses, remainder in-game purhases in gaming)	Some reporting on sleep problems, academic problems, mental illness, significant life events, interpersonal problems	n/a
Bensimon et al. (63)	Israel	In-depth interviews with patrons (*N* = 10) and staff (*N* = 4) at an illegal casino	Schnitzer Foundation for Research on the Israeli Economy and Society	Attempted suicide at the casino premises after incurring heavy gambling debts.	n/a	n/a
Blaszczynski and Farrell (36)	Australia (Victoria)	Coroner’s reports on completed suicides 1990–1997 with reference to gambling (*N* = 44).	n/a	All the gamblers had financial difficulties prior to suicide with heavy debts and losses. In 8 cases also criminal acts to finance gambling.	Depression (32 percent), alcohol abuse (14 percent), alcohol and drug abuse (6 percent), drug abuse (4 percent). Relationship difficulties (34 percent), work problems (9 percent). Personality characteristics such as introversion and low self-esteem.	25 percent had sought help, but attendance was mostly low and appointments were not kept.
Gill et al. (61)	Global	Public records of case studies on suicide in the absence of a psychiatric disorder (*N* = 10)	n/a	In each case, gambling or gambling debts were identified as the cause of suicide or familicide.	None. One of the aims of the paper is to explore suicidality without mental disorder.	n/a
Jacobs (64)	United States	Case study of a 52-year old suicidal man with a history of gambling	n/a	Gambling debts described as the direct cause of suicidality. Lack of remarkable medical history.	Possibility of depression, terminal illness of a family member	Gambling addiction program
Kaggwa et al. (56)	East African community	Press reporting on gambling-related suicides (*N* = 18)	None	The most common identified type of suicide consisted of university students losing their tuition fees on gambling and committing suicide.	Possibly high levels of poverty.	n/a
Kausch et al. (52)	United States (Ohio)	Treatment seeking veterans with diagnosed pathological gambling (*N* = 111), including three detailed case reports	n/a	n/a	Selection criteria included individuals who had experienced trauma, including various types of abuse in childhood and adulthood. Also substance abuse	n/a
Kizza et al. (67)	Uganda	Qualitative psychological autopsy of men who died by suicide (*N* = 17)	None	In one case, gambling losses and related shame appear to have driven the individual to suicide.	Poverty and unemployment identified behind gambling as well as suicide.	n/a
Komoto (40)	Japan	Case study of a woman with a 13 year history of diagnosed pathological gambling.	The Nikkoso Foundation for Safe Society (Japan)	Gambling described as a consequence of feelings of guilt. Gambling worsened the psychological situation of the individual, resulting in a suicide attempt.	Depression and feelings of guilt	CBT as well as Naikan therapy to diminish feelings of guilt. Following treatment the patient has been able to abstain for 1 year.
Leung et al. (54)	Hong Kong	Interview study with individuals with a history of self-harm (*N* = 11).	n/a	Pathological gambling identified as one of the main contributing factors for self-harm.	Family problems	Preference for specialized gambling services.
Luquiens et al. (58)	France	Quantitative and qualitative observations based on contacts to gambling and gaming helpline during a 7-year period (*N* = 14,564)	None	Reports of contemplating suicide due to gambling debts.	Gamblers reported suicidal thoughts as a result of psychological exhaustion and lack of help.	n/a
Matthew and Volberg (65)	Singapore	In-depth interviews with CSOs of problem gamblers (*N* = 50)	Ministry of Community, Youth and Sports (Singapore)	Family members threaten suicide if they do not receive money from their CSOs for gambling/debts.	n/a	n/a
Miller et al. (62)	Australia	Press reporting on problem gambling in 2011–2012 (*N* = 339)	n/a	Twenty articles (6%) discussed suicide. Suicide was attributed to heavy gambling and related debts.	Financial debt, depression	Control on gambling availability suggested as a preventive strategy.
Oakes et al. (66)	Australia (South Australia)	Interviews with problem gamblers, CSOs and service providers (*N* = 54)	Flinders University Faculty of Social Sciences (Australia); Gambling Research Australia (GRA)	Shame and harms associated with gambling as a cause for suicidal thoughts for people gambling problematically. Suicide as a means to escape a desperate situation.	n/a	Suicidal thoughts as rock bottom that make the individual seek help
Reith and Dobbie (68)	UK	Interviews with a cohort of 50 gamblers interviewed three times between 2006–2009	UK Economic Social Research Council; The Responsibility of Gambling Trust (now The Responsible Gambling Trust), UK	Shame and exhaustion due to gambling identified as causes for suicidal ideation	n/a	Suicidal thoughts or attempted suicide as rock bottom that made the individual seek help and project themselves in the future.
Sharma et al. (60)	India	Case report of a diagnosed pathological gambler.	None	Heavy gambling debts, guilt, pressure by CSOs to stop gambling preceded the suicide attempt	Co-morbid depression was diagnosed, but the onset of depression	
					was identified as having taking place after problematic gambling. Impulsive personality.	A failed suicide attempt made the individual seek help. CBT, Motivational enhancement therapy and medication appear to have been effective in maintaining abstinence for 3 months.
Sichali et al. (59)	Malawi	Narrative case report of a young individual who committed suicide after losing money on gambling.	Wellcome Trust (UK)	Gambling losses and subsequent conflicts identified as the main reason for the suicide	n/a	Suggested challenging the responsible gambling paradigm.
Wong et al. (28)	Hong Kong	Psychological autopsy interviews concerning gambling-related suicide cases (*N* = 17)	Hong Kong Jockey Club Charities Trust; The National Institute of Mental Health (NIMH), US	In each identified case, the suicide victim had unmanageable debt at the time of death.	The most common associated psychiatric disorder of suicide victims was depression. However, problematic gambling appears to have preceded depression.	n/a

The relationship between gambling and suicide or suicidality, as well as how gambling contributes to suicide was charted using three analytical categories: (1) contribution of gambling to suicide or suicidality; (2) confounding factors; (3) support or recovery. We also noted additional observations. To limit the impact of individual bias in interpretation, two separate researchers reviewed the original studies and agreed on the topics and results of the analysis. Ethics approval was not required for the study as it based on existent and publicly available research evidence.

## Results

Overall, the quantity of papers included in this study show that there is a scarcity of qualitative research on gambling-related suicides and suicidality. However, geographically this type of research is quite well-spread. The studies included in this review represented a wide geographical distribution, including several contexts in Europe, Asia, Africa, Australia, and North America.

In line with the PRISMA guidelines (51) we also noted the funding sources for all the included studies. In the case of eight (8) studies, the information was not available (n/a). Given that industry funding has been an issue for example in the case of tobacco research, funding declarations would be important.

### How gambling and gambling-related debt contributes to suicides and suicidality

As per the inclusion criteria, all the included studies (*N* = 20) discussed how gambling contributed to suicides or suicidality. In the vast majority of these studies (19/20), gambling or gambling-related harms were seen as the main reason for a suicide. In only one study (52), gambling was rather seen as a coping mechanism to deal with other trauma in life that was also identified as the main reason for suicidality. However, the aim of the study in question was to investigate individuals with trauma which may explain the diverging perspective.

In some studies (53–55), gambling itself was described as the generic cause of suicide. Leung et al. (54) interviewed suicidal or self-harming individuals and found that pathological gambling was identified as one of the main contributing factors for self-harm. A case study of an individual in Estonia having committed familicide (55) found that the person had not exhibited suicidal behavior before commencing heavy gambling on electronic gambling machines (EGMs).

Other research identified two main processes through which gambling appears to contribute to suicidality.

The first, and most prevalent, of these processes related to indebtedness and money loss. Gambling-related debt or significant losses of capital were identified as a cause or at least an important contributor to suicide or suicidality in several studies (28, 36, 53, 56–64). This was true of both studies focusing on suicide victims more generally and of studies focusing on gambling specifically. For example, Miller et al. (62) studied press items on pathological gambling in Australia and found that in articles discussing suicide (*N* = 20), it was attributed to heavy gambling and related debts. Another study using press reporting on gambling and gaming related suicides in India (53) found that 83 percent of all reporting linked suicide to financial loss due to gambling or gaming. A study using press reporting on gambling-related suicides in East Africa (56) similarly found that in the most typical case, the victim was a university student having lost their tuition fees on gambling prior to committing suicide.

Psychological autopsy studies or research using coroner’s reports on completed suicides have similarly found indebtedness and heavy losses prior to death. For example, a study on gambling-related suicides in Hong Kong (28) found that in each identified case, the suicide victim had unmanageable debt at the time of death. Another study on completed suicides in Victoria, Australia (36) found that all the gamblers had heavy debts and losses before committing suicide. Some had also resorted to criminal acts to finance their gambling. Case studies from Malawi (59) and India (60) similarly show that gambling losses and subsequent family conflicts were the main reason behind the described suicide or suicide attempt.

Besides completed suicides, indebtedness is also often reported as a reason for contemplating suicide. As described by Luquiens et al. (58) based on data collected from contacts to a gambling helpline, heavy debts can make the individual feel like suicide is the only solution. If the debts have been incurred due to illegal gambling, this may put further stress on the individual to seek a desperate solution as described by Bensimon et al. (63) in a study of gamblers at an illegal casino in Israel or (64) in a case report of a suicidal individual in the United States. In some cases, suicide can also be used as a threat. Matthew and Volberg (65) interviewed concerned significant others of gamblers in Singapore and found that gambling family members may threaten suicide if they do not receive money from their family members for gambling or gambling-related debts.

The second process by which gambling appears to be connected to suicidality relates to shame. Several studies described how gambling either created or worsened feelings of shame and guilt, eventually leading to suicide or a suicide attempt (40, 60, 66–68). In one case study from Japan (40), the individual was described to have already fostered feelings of shame and guilt before commencing gambling. However, gambling worsened the psychological situation, resulting eventually in a suicide attempt. In other studies, the feelings of shame have rather resulted from excessive gambling or gambling losses (67, 68). One study, based on interviews with gamblers, their significant others, and service providers (66), found that shame associated with gambling was seen as an important contributing cause for suicidal thoughts for people gambling problematically. Suicide then becomes a means to escape the difficult feelings as well as a desperate situation.

### Role of confounding factors

In our material, 14 out of the 20 studies included discussion on confounding factors or co-occurring diagnoses that may have also contributed to suicidal behavior. Based on the data, these factors can be divided into three main categories related to psychiatric comorbidities, personality types, and life situation.

Regarding psychiatric comorbidities, depression (or more generic mental disorder) was cited in several studies (28, 36, 40, 53, 55, 60, 62, 64). Alcohol or drug abuse was also cited in some studies (52); Blaszczynski and Farrell (36) found in their study of coroner’s reports in Australia that 32 percent of gambling-related suicide victims had depression. Fourteen percent had alcohol abuse, six percent abused both alcohol and drugs, and four percent abused drugs only. However, in three studies in which the temporal order between depression and gambling was investigated, the analysis found that both depression and suicidality had emerged only after gambling-related problems (28, 55, 60). Luquiens et al. (58) also found that gamblers reported suicidal thoughts as a result of psychological exhaustion and lack of help for gambling problems.

Not all studies found other co-morbid psychiatric diagnoses. For example, a historical collection of case studies presented by Gill et al. (61) showed that gambling-related suicide can and does also occur without mental disorders.

Personality characteristics were mentioned as possible confounding factors in a few studies. Notably, impulsive personality traits were mentioned in case studies of suicide victims (55, 60). Blaszczynski and Farrell (36) also found references to introversion and low self-esteem in Australian coroner’s reporting.

The most often mentioned confounding factors were related to other trouble or hardship in life and life situation. Some of these problems had preceded gambling. Notably studies describing gambling-related suicides in Africa showed that high levels of poverty as well as unemployment had contributed to gambling (56, 59, 67). Kizza et al. (67) describe in their psychological autopsy study of suicides in post-conflict Uganda that many individuals started gambling due to otherwise limited opportunities of income. In other research, family history of suicide, or trauma, such as various types of abuse in childhood and adulthood, were identified as triggering factors to gambling and eventually suicidality (52, 64).

Other life situation related issues appear to have rather stemmed from gambling or emerged after gambling had commenced. These related notably to interpersonal problems and relationship difficulties (36, 53, 54) or to problems at study or work (36, 53). These are typically related to gambling debts and losses [also Blaszczynski and Farrell (36)].

### Treatment and recovery

We also noted any mentions of treatment for and recovery from suicidality. However, only eight studies discussed these issues. Based on the observations, the main motivation for individuals to seek help appears to have been “hitting a rock bottom”; Suicidal thoughts or suicide attempts were described as such a low point in several studies (64, 66, 68, 69). For example, Kizza et al. (67) describe a case in which suicidality allowed the individual to start projecting themselves in the future and therefore to finally seek help for their gambling.

The types of treatment received were also discussed in a few of the included studies (36, 40, 54, 55, 69). Although observations are not many, findings suggest that the more comprehensive and specialized the treatment and support approach, the better the results. Notably, a case study from Japan shows that cognitive behavioral therapy (CBT) combined with Naikan therapy to diminish feelings of guilt had resulted in abstinence from gambling for a year. In a case study from India (60), CBT combined with motivational enhancement therapy and medication had been effective in maintaining abstinence from gambling and suicidality for 3 months. Elsewhere, approaches based on only medication (55) or appointments that were often not kept (36) were not effective.

In addition to treatment and support, some studies also discussed how suicidal behavior could be prevented in the first place. For example, Miller et al. (62) noted based on their study on gambling-related problems in Australian press reporting, that there needs to be a better control of gambling availability and better controls on gambling to prevent excessive engagement in gambling and related suicidality. The case study of a suicide in Malawi (59) also concluded that the so-called responsible gambling paradigm puts blame for gambling problems on individuals and functions as a barrier to seek help. Challenging this paradigm would therefore be helpful in preventing individuals from committing suicides due to gambling and related harms.

## Discussion

Based on the results of the current review, gambling can be identified as an important contributor to suicide. The qualitative research evidence summarized in this study indicates that the two main processes that connect gambling to suicide or suicidality are indebtedness and shame. These two processes have also been identified in previous research literature, and gambling has been found to be a significant contributing factor to unmanageable indebtedness as well as to crippling shame (4, 29, 70).

The results of the current study are in line with the interpersonal theory of suicide (71). The theory has found that perceived burdensomeness and lack of belonging contribute to a desire for suicide. Both processes can also be linked to indebtedness and shame: Unmanageable debt and feelings of shame can be seen as hindrances for interpersonal interaction and belongingness.

At the same time, suicide is a complex and multifactorial phenomenon, limiting the determination of direct causality. Other confounding factors are also present for some, consisting notably of psychiatric comorbidities. In most cases, both comorbidities (notably depression) as well as other difficulties (relationship and work problems) appear to have emerged as consequences of gambling. There is also evidence that heavy indebtedness alone without other comorbidities appears to contribute to suicidality (29). This finding somewhat contrasts with evidence suggesting that depression rather than gambling is the causal reason behind suicide (34, 72). Quantitative suicide research on risk factors beyond the gambling field (73); also Hjelmeland and Knizek (48) has shown that a causal link between depression and suicide is typically accepted and implied. However, as argued by Hjelmeland and Knizek (48) this may also be due to methodological issues, lack of qualitative evidence, and publication bias. Furthermore, even in cases where a direct causality cannot be determined, it is fair to say that gambling has negatively affected the individual.

Treatment and support appear to have been effective in some cases, but there are also important barriers to seek and receive treatment. Resources and commitment to appointments may be one [cf. Blaszczynski and Farrell (36) and also Séguin et al. (74)], suggesting that additional engagement in treatment is necessary. At the same time, the processes of shame and indebtedness may also work as barriers. As also noted by Hing et al. (70) deep feelings of shame and self-stigma result in secrecy and unwillingness to seek help. Similarly, Dufour and Roy (5) have found that individuals may wish to conceal the extent of their indebtedness from others, and therefore do not seek help. Making the situation worse, particularly in the case of indebtedness, continued gambling is not only the cause but also the perceived solution to financial problems (5). The actual processes that connect gambling to suicide and suicidality therefore also appear to function as barriers to helping these individuals.

The only way to effectively prevent extreme gambling-related harms, such as suicidality, and the processes of indebtedness and shame that connect to it, appears to be shifting perspective in regulation toward a more comprehensive public health approach and prevention at a population level to relieve the blame on individuals. Gambling has become widely accessible across the world. Growing gambling markets in the global south, such as Africa (75), are also visible in the increasing attention that has been paid to gambling-related suicides in these contexts in recent years. In the more mature gambling markets of the global north, issues such as the responsible gambling mantra has been argued to increase shame for those who are supposedly irresponsible with their gambling (76). The normalization of gambling, easy accessibility, and individual responsibility rather than controls on industry actors increase total consumption on gambling and therefore also total harms. These harms are not limited to problem gambling prevalence, but to a wide array of intertwined harms to individuals, communities, and societies (77, 78).

The current study has been limited to a restricted sample of studies, due to the overall lack in qualitative evidence on gambling-related suicides and suicidality. The complexity of the suicide phenomenon also limits the possibilities to draw linear cause and effect conclusions. While we have identified two key processes that connect gambling and suicide, there is still need for more research to ascertain these processes and to investigate other possible “pathways.” Notably, there is need for more research on how individuals understand the meaning of suicide, and how suicidality can be placed within a person’s lived experience (48). For example, socio-cultural contexts and gender differences may impact lived experience and risk for suicidal behavior (47, 48). Due to the limited number of studies analyzed in the current review, these types of issues could not be systematically analyzed but should be addressed in future studies.

To prevent gambling-related suicidality in the future, a public health approach is needed to shift the blame for gambling-related harms and indebtedness from often vulnerable individuals to the societal and commercial conditions (79–81). At the same time, it is crucial to target the speed of gambling products, advertisement of gambling particularly to the young (82), but also put an end to easy and fast access to credit that enables debt-driven gambling.

## Data availability statement

The original contributions presented in this study are included in the article/supplementary material, further inquiries can be directed to the corresponding author.

## Author contributions

VM conducted the analysis and wrote the first draft of the manuscript. Both authors were in charge of data collection and the conception of this manuscript, revised the draft, and gave their final approval.
